# Microcytosis in Erythropoietic Protoporphyria

**DOI:** 10.3389/fphys.2022.841050

**Published:** 2022-03-03

**Authors:** Giovanna Graziadei, Lorena Duca, Francesca Granata, Giacomo De Luca, Anna De Giovanni, Valentina Brancaleoni, Isabella Nava, Elena Di Pierro

**Affiliations:** ^1^Dipartimento di Medicina Interna, Fondazione IRCCS Cà Granda Ospedale Maggiore Policlinico, Milan, Italy; ^2^Università degli Studi di Milano, Milan, Italy

**Keywords:** erythropoietic protoporphyria, PPIX accumulation, microcytosis, iron and heme deficiency, anemia, hepcidin

## Abstract

Partial deficiency of the last enzyme of the heme biosynthetic pathway, namely, ferrochelatase (FECH), is responsible for erythropoietic protoporphyria (EPP) in humans. This disorder is characterized by painful skin photosensitivity, due to excessive protoporphyrin IX (PPIX) production in erythrocytes. Although several papers report the presence of iron deficiency anemia in about 50% of EPP patients, there is still no a conclusive explanation of the why this occurs. In the present work, we explored hematological indices and iron status in 20 unrelated Italian EPP patients in order to propose a new hypothesis. Our data show that microcytosis is present in EPP patients also in the absence of anemia and iron deficiency with a link between PPIX accumulation and reduced MCV, probably indicating an indirect condition of heme deficiency. Patients studied had a downward shift of iron parameters due to increased hepcidin concentrations only in a state of repleted iron stores. Interestingly, hemoglobin synthesis was not limited by iron supply except in cases with further iron loss, in which concomitantly increased soluble transferrin (Tf) receptor (sTfR) levels were detected. The mechanisms involved in the iron uptake downregulation in EPP remain unclear, and the role of PPIX accumulation in microcytosis.

## Introduction

Erythropoietic protoporphyria (EPP, MIM 177000) is a rare inherited disorder caused by the deficiency of ferrochelatase (FECH; EC4.99.1.1) that catalyzes the chelation of ferrous iron by protoporphyrin IX, in the last step of the heme biosynthetic pathway (Lecha et al., 2009; Balwani, 2019). EPP occurs because of a loss-of-function mutation in one allele of the *FECH* gene, which is primarily inherited in trans to a low expression *FECH* allele carrying common genetic variants (c.1 –252G, c.68–23T, c.315–48C), causing a 70% reduction in enzymatic activity (Gouya et al., 2002; Chiara et al., 2020). FECH deficiency leads to significantly elevated protoporphyrin IX (PPIX) levels mainly in erythrocytes and subsequently in the skin and liver (Balwani et al., 2017; Di Pierro et al., 2022). The PPIX accumulation causes lifelong acute photosensitivity with childhood-onset and several degrees of hepatobiliary involvement with acute liver failure occurring in 1–5% of patients (Anstey and Hift, 2007; Wensink et al., 2021). In contrast to defects arising in other genes implicated in mitochondrial iron use, such as *ALAS2* and *ABCB7*, abnormal iron deposits are not observed linked to FECH abnormalities (Karim et al., 2015). On the contrary, mild hypochromic, microcytic anemia has been reported in 33% of men and 48% of women with EPP (Holme et al., 2007) although, the explanation of the why this occur remains unclear. Indeed in EPP patients was observed a two-third decrease in the iron stores, assessed by serum ferritin. However, serum iron concentrations and soluble transferrin receptor-1 (sTfR), a marker of iron deficiency and ineffective erythropoiesis, have been reported to be normal, confirming the iron is sufficient for hemoglobin synthesis in EPP (Holme et al., 2007; Delaby et al., 2009). Moreover, the levels of Hepcidin, the primary iron metabolism regulator, were not observed to be inappropriately increased in both serum and urine excluding the hypothesis of a lack in the iron absorption (Bossi et al., 2015). Finally, the iron depletion in EPP does not appear to be related to chronic inflammation (Barman-Aksoezen et al., 2017). Although the mechanisms explaining how iron is kept low in EPP remain to be identified, there is accumulating evidence that iron deficiency can mitigate disease expression of EPP (Poli et al., 2021). In the bone marrow, heme synthesis is mainly controlled by the intracellular labile iron pool by post-transcriptional regulation. Indeed, translation of ALAS2 mRNA, the first and rate-limiting enzyme of the heme biosynthesis pathway in the erythroid tissue, is inhibited when iron availability is low by the 5’UTR IRE-IRP system regulation (Wilkinson and Pantopoulos, 2014; Silva and Faustino, 2015). At the same time, it has been shown that the FECH enzyme activity is directly regulated by the availability of the iron–sulfur [2Fe-2S] cluster contained at the C-terminus of the enzyme (Taketani et al., 2000). Moreover, an increase in aberrant *FECH* transcripts was reported in iron-depleted cells (Barman-Aksozen et al., 2013). Then, iron deficiency might lead to less PPIX accumulation by inhibiting ALAS2 but should also increase PPIX level by inhibiting FECH action (Buzzetti et al., 2022).

Concerning these contrasting observations this study aimed to evaluate anemia and the iron status in EPP patients cared at the Rare Disease Centre at Fondazione Ca′ Granda Policlinico of Milan in order to provide new suggestions for the open questions.

## Materials and Methods

### Patients and Controls

Twenty Italian unrelated EPP patients and 20 sex and aged-matched healthy volunteer individuals were included in the study before starting oral iron supplementation if necessary. The EPP patients, 11 males (34 ± 10 years) and nine not pregnant females (37 ± 10 years), all exhibited a typical plasma fluorescence peak at 632–635 nm and an increase of total erythrocyte protoporphyrin (ePP) concentrations with predominance of free protoporphyrin (PPIX; Di Pierro et al., 2021). In addition, all patients had a classical EPP genetic diagnosis with a loss-of-function FECH mutation in trans to the hypomorphic allele. Table 1 reports the biochemical and genetic characteristics of the patients. Only the patient n.13 carried a questionable pathogenetic variant (c.163G > T); however, the presence of a large deletion in the gene cannot be excluded for this patients. Furthermore, all included patients showed no elevation of hepatic necrosis and stasis indices and inflammatory parameters. The study was conducted following the Declaration of Helsinki for medical research.

**Table 1:** Biochemical and genetic data of the EPP patients.

	Sex	Age	PPIX (μg/gHb)	Zinc-PP (μg/gHb)	*FECH* genotype
Pt 1	M	32	121,5	2,5	c.[1-251G > C;194 + 4350_463 + 1197del5577];[315-48T>C] Ref.: Di Pierro (2006) Hum Genet 118,776
Pt 2	F	29	64,3	6,4	c.[215dupT];[315-48T > C] Ref.: Wang (1997) J Invest Dermatol 109,688
Pt 3	M	26	75,3	2,3	c.[1-251G > C;194 + 4350_463 + 1197del5577];[315-48T > C]
Pt 4	M	31	119,6	2,4	c.[215dupT];[315-48T > C]
Pt 5	F	41	64,4	3,4	c.[901_902delTG];[315-48T > C] Ref.: Schneider-Yin (1994) Hum Genet 93, 711
Pt 6	F	48	168,8	5,2	c.[901_902delTG];[315-48T > C]
Pt 7	F	32	87,3	3,6	c.[215dupT];[315-48T > C]
Pt 8	M	48	65,7	3,5	c.[464-1169A > C];[315-48T > C] Ref.: Chiara (2020) Genet Med 22, 35
Pt 9	M	21	38,1	2,9	c.[215dupT];[315-48T > C]
Pt 10	F	24	27,7	2,7	c.[215dupT];[315-48T > C]
Pt 11	F	50	46,6	2,5	c.[464-1169A > C];[315-48T > C]
Pt 12	M	21	64,5	2,7	c.[67 + 5G > A];[315-48T > C] Ref.: Wang (1999) J Invest Dermatol 113, 87
Pt 13	M	33	130,3	2,7	c.[163G > T];[315-48T > C] Ref.: Lamoril (1991) Biochem Biophys Res Commun 181, 594
Pt 14	M	37	92,6	1,9	c.[215dupT];[315-48T > C]
Pt 15	F	23	48,3	3,1	c.[1080_1081delTG];[315\u201348 T > C] Ref.: Ventura P. (2020) Eur J Dermatol;30(5):532–540.
Pt 16	F	46	64,3	3,4	c.[215dupT];[315-48T > C]
Pt 17	M	36	127,1	2,6	c.[215dupT];[315-48T > C]
Pt 18	M	44	28,1	5,0	c.[464-1169A > C];[315-48T > C]
Pt 19	M	50	119,1	3,7	c.[215dupT];[315-48T > C]
Pt 20	F	43	29,3	2,5	c.[215dupT];[315-48T > C]

*PPIX are free protoporphyrins, n.v.:0.0–3.0 μg/gHb. Zinc-PP are zinc chelated protoporphyrins, n.v.: 0.0–3.0 μg/gHb. FECH genotype is reported according to the HGVS nomenclature (version 20.05) based on NM_000140 transcript; the reference of the first description of each pathogenic variant is also indicated*.

### Hematologic and Biochemical Measurements

Venous blood collection in both non-anticoagulated and EDTA vacutainers was obtained from EPP patients during winter. Blood samples were examined for standard hemochrome-cytometric parameters and iron status. Among the studied hematological parameters were total hemoglobin (Hb) and mean corpuscular volume (MCV). Serum iron was determined using standard colorimetric methods; serum transferrin was determined using the turbidimetric method; the percentage of transferrin saturation (TfS%) was calculated using a correction factor (1.4). Serum ferritin was measured by a standard enzyme immunoassay method. Ranges of normal values were obtained from routine analysis of the general Italian population. An additional non-anticoagulated vacutainer was collected from both EPP patients and healthy subjects. After centrifugation for 15 min at 3000 rpm sera fractions were collected and stored at −80° C for the following analysis. The soluble transferrin receptor (sTfR) was measured by immunoenzymatic ELISA using BioVendor (Research and Diagnostic Products, Karasek, Czech Republic); the test is based on a non-competitive “sandwich-type” assay technique. Serum Hepcidin-25 was determined by competitive immunoassay (EIA) using Bachem (Peninsula Laboratories International, San Carlo, CA, United States). Antibodies coated on a 96-well plate captured the antiserum. The addition of a constant concentration of biotinylated tracer competed for the specific binding. Streptavidin conjugated horseradish peroxidase bound the biotinylated complex producing a colored solution, upon addition of a substrate, quantifiable at 450 nm with a plate spectrophotometer. Normal reference values were referred to control subjects included in the study for these two analyses.

### Statistical Analysis

The normal distribution of continuous variables was analyzed using the D’Agostino–Pearson’s and Shapiro–Wilk normality tests. Means, medians, standard deviation (SD), interquartile range (IQR) have been calculated when appropriate. The parametric and non-parametric tests (ANOVA and the Mann–Whitney tests, respectively) were employed to compare groups. Pearson’s correlations were determined between two data sets with a two-tailed test and confidence interval at 95%. Linear regression was also calculated at a confidence interval of 95%. Values of *p* lower than 0.05 were considered statistically significant. The data analysis was performed using GraphPad Prism software (version 9.0, Inc., United States).

## Results

### Microcytosis Without Anemia and Iron Deficiency in EPP Male Patients

In all patients (11 males and nine females) the values of hemoglobin (Hb), mean corpuscular volume (MCV), serum iron, ferritin, transferrin (Tf) were evaluated and the percentage of saturation (TfS%) was calculated. 55% of patients, 7/11 males (64%) and 4/9 females (44%), had mild microcytosis, defines as MCV < 80 fL in males and <78 fL in females. However, only female patients presented mild anemia while male patients presented microcytosis despite normal Hb levels (Table 2). Serum transferrin and iron concentrations were normal in all patients. However, in 7/9 females and 2/11 males, serum ferritin and TfS% were reduced. Among these, three females had neither anemia nor microcytosis and the two males did not show anemia.

**Table 2:** Hematological and iron status examinations in EPP patients classified according to gender and microcytosis.

	Hb (g/dL)	MCV (fL)	Iron (mcg/dL)	Tf (mg/dL)	TfS (%)	Ferritin (ng/mL)	sTfR (mcg/mL)	Hepcidin (ng/mL)
Normal female reference values	12–16	78–99	37–145	200–360	18–24	15–150	0.67–1.78	6–22
Normal male reference values	13.5–17.5	80–94	59–158	200–360	26–35	30–400	0.58–1.72	6–21
Microcytic EPP females (4/9)	11.2 ± 0.41^*^	75.0 ± 1.48	44 ± 14^*^	309 ± 17	12 ± 2^*^	7 ± 2^*^	2.95 ± 0.98^°^	3.84 ± 3.78
Microcytic EPP males (7/11)	14.3 ± 0.15	75.9 ± 0.99	100 ± 36	302 ± 24	29 ± 5	47 ± 9	1.97 ± 0.41	20.5 ± 5.01
Normocytic EPP females (5/9)	12.6 ± 0.23	83.6 ± 1.25	70 ± 42	300 ± 38	20 ± 5	21 ± 7	2.14 ± 0.78	25.9 ± 9.21
Normocytic EPP males (4/11)	15.2 ± 0.38	83.1 ± 1.20	126 ± 45	298 ± 38	32 ± 5	97 ± 34	1.59 ± 0.75	50.4 ± 16.6

*Hemoglobin [Hb], mean corpuscular volume [MCV], transferrin [Tf], Tf saturation [TfS], serum Tf receptor [sTfR]. Data are expressed as mean ± SD. All reference values were obtained from routine analysis of Italian general population except for sTfR and Hepcidin for which the normal ranges were obtained from control subjects included in the study.*

* *p = 0.008 microcytic females vs microcytic males.*

° *p = 0.05 microcytic females vs microcytic males.*

### Iron Deficiency With Preserved Iron Metabolism Regulation

**Figure 1:**
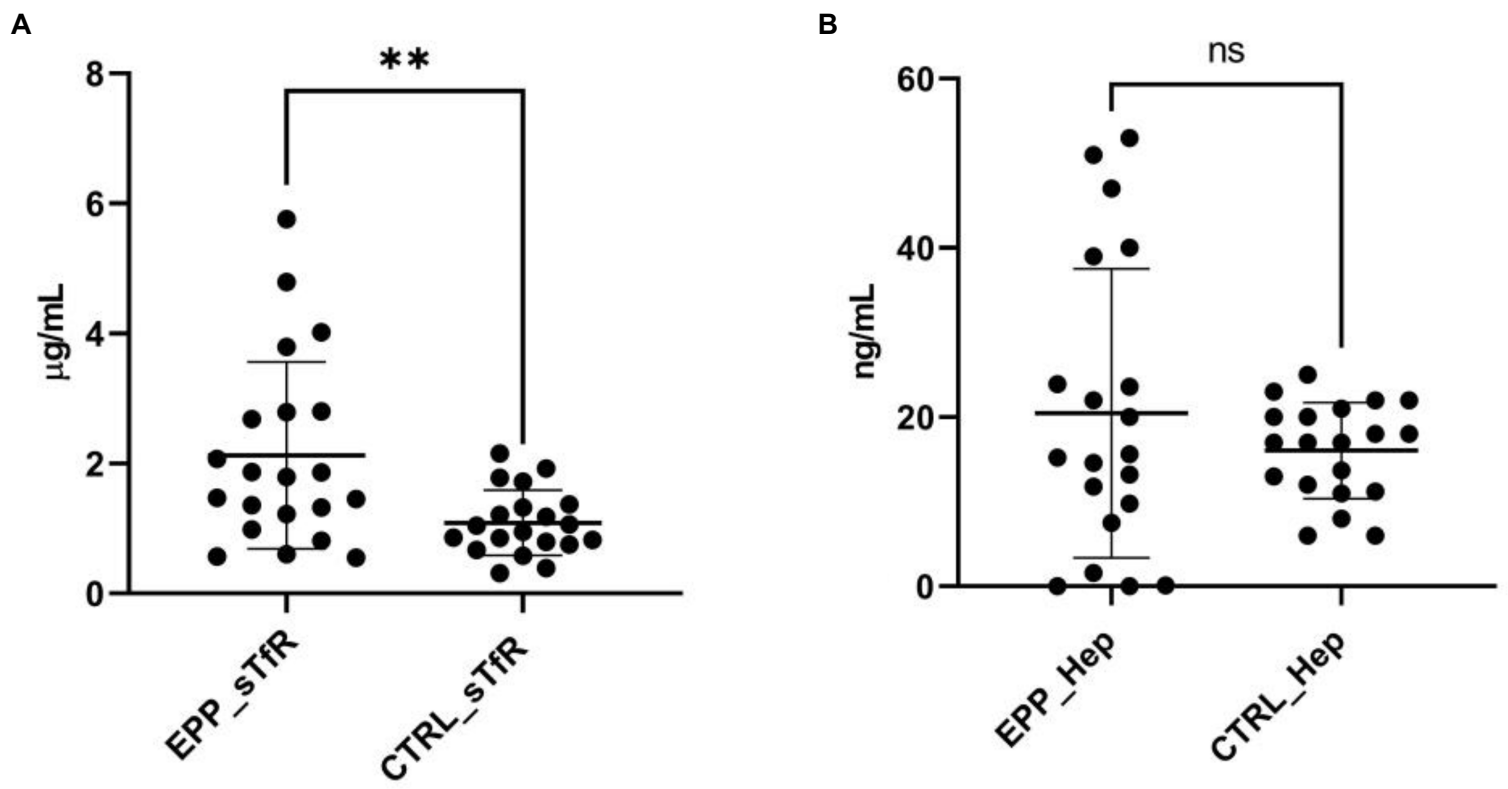
sTfR and hepcidin measurements. **(A)** sTfR distributions in EPP patients compared to healthy subjects (CTRL); **(B)** hepcidin distributions in EPP patients compared to healthy subjects (CTRL). **p < 0.01, ns = not significant.

We then explored further iron parameters. The mean values of serum soluble transferrin receptor-1 (sTfR) concentrations were significantly increased in EPP patients compared to healthy subjects (Figure 1A). Moreover, although a trend of rising values of hepcidin was observed, no significant difference was detected between EPP patients and healthy subjects (Figure 1B).

**Figure 2:**
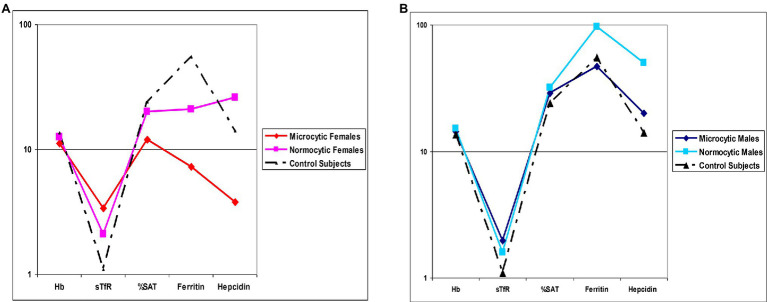
Mean values comparison. **(A)** Hematological and iron status parameters in microcytic and normocytic females compared to Controls **(B)** Hematological and iron status parameters in microcytic and normocytic males compared to Controls. Hemoglobin [Hb], serum Tf receptor [sTfR], Tf saturation [%SAT].

**Figure 3:**
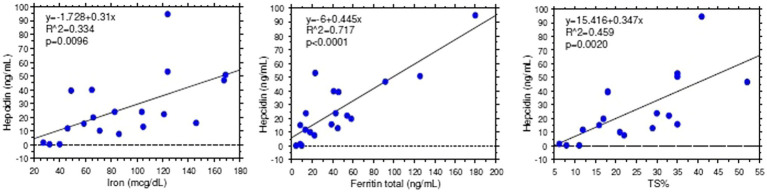
Hepcidin vs. iron parameter correlations.

Stratification of patients based on gender and microcytosis showed that sTfR mean values were slightly increased in the male EPP patients compared to healthy subjects while higher values were observed in females, particularly in microcytic ones with reduced TfS% and ferritin (Figure 2A). Unexpectedly, hepcidin levels were higher than normal in all normocytic patients, normal in microcytic males, and lower in microcytic anemic females (Figure 2B). However, hepcidin levels directly correlated with iron (r2 = 0.334; *p* < 0.001), ferritin (*r*^2^ = 0.717; *p* < 0.0001) and TS% (*r*^2^ = 0.459; *p* = 0.002) as expected in cases in which the iron regulation mechanism is properly maintained (Figure 3).

### Microcytosis and Protoporphyrin IX Correlation

There was no significant correlation among iron, ferritin, TS%, and MCV while an inverse correlation was detected between MCV and PPIX (*r*^2^ = 0.297, *p* = 0.013; data not shown). In addition, all microcytic patients showed PPIX mean levels significantly higher than normocytic patients (Table 3).

**Table 3:** Protoporphyrin IX values in microcytic and normocytic EPP patients.

	Mean	SD	SE	Min	Max	Sum	Median	IQR
Microcytic EPP females	96.3^^^	49.6	25	64	169	385	76	44–108
Microcytic EPP males	104.4^*^	26.7	10	65	130	731	120	97–143
Normocytic EPP females	43.2	15.0	7	28	64	216	47	35–59
Normocytic EPP males	62.8	40.8	20	28	119	251	52	22–82

*The values of protoporphyrin IX are expressed as μg/gHb.*

^ *p = 0.0304 microcytic vs normocytic EPP Females.*

* *p < 0.0001 microcytic vs normocytic EPP Males*.

## Discussion

FECH is the last enzyme of the heme biosynthetic pathway and it is deputed to the insertion of iron into protoporphyrin IX (PPIX). Although high levels of PPIX accumulate because of reduced FECH activity, no signs of iron overload are found in patients with EPP. On the contrary, it is estimated that 20%–60% of EPP patients develop mild microcytic anemia whose pathogenesis is unclear. Among the 20 patients of this study, we observed that 7/11 males (64%) and 4/9 females (44%), had a mild reduction in the MCV values. However, only female patients presented anemia while male patients presented microcytosis despite normal Hb and iron levels (Table 2). Moreover, 45% of patients (7 females and two males) showed reduced serum ferritin levels and TfS% but not all of them developed microcytic anemia. As reported by previous works (Holme et al., 2007), our results confirm that EPP patients tend to have lower serum iron, ferritin, and transferrin saturation levels than normal even if within normal ranges. This could make them more susceptible to developing anemia in blood loss cases. Blood loss is mainly intestinal or in females due to menstruation and pregnancy (Ganz, 2013). Indeed, in our cohort, only females, all in childbearing age and known to have iron loss with abundant menstrual cycles, showed anemia. On the contrary, the observation that microcytosis occurred in males without iron depletion suggests that other mechanisms other than iron deficiency could be involved in this hematological feature in EPP. This hypothesis is also supported by the fact that iron deficiency anemia similarly occurs in EPP to that in the healthy population in which serum ferritin <12 μg/L indicates depleted iron stores and results in reduced hemoglobin concentration (Cook and Finch, 1979; Skikne et al., 1990).

Moreover, in contrast to Holme et al., we found a significant increase in serum sTfR levels in EPP patients. sTfR concentrations are usually increased under two conditions associated with increased iron absorption, that is, iron deficiency and increased erythropoiesis. However, the levels of GDF15, a marker of erythropoietic activity (Tanno et al., 2010), were reported to be normal in EPP, excluding the presence of erythropoietic stress (Barman-Aksoezen et al., 2017). Then, we can conclude that EPP patients have an intrinsic state of tissue iron deficiency. Indeed, we also observed that the reduction in ferritin levels was not always accompanied by a decrease in iron blood levels and anemia. This could suggest that iron in EPP, although limited, can be in any case sufficient for erythropoiesis. In this state of equilibrium, the absorption and availability of iron could be commensurate with the reduced ability of the FECH enzyme to incorporate iron into PPIX. This would prevent iron accumulation in the body, the substrate with potentially more significant toxicity.

In accordance with previous works, we show that the iron regulation mechanism in these patients is not disrupted (Bossi et al., 2015). Indeed hepcidin, the key regulator of iron homeostasis in humans, moves up and down in an inverse manner to the iron parameters as expected (Ganz, 2013). Hepcidin acts reducing the display and expression of ferroportin on the plasma membranes of enterocytes and macrophages. Thus, higher hepcidin levels lead to the internalization of ferroportin and to decreased release of iron from enterocytes into the portal blood and from macrophages into the blood (Fleming and Sly, 2001). In this study, levels higher than average have been observed in EPP normocytic patients with normal iron stores. This suggests that some signals may stimulate hepatic hepcidin synthesis to keep iron levels low in EPP. These results disagree with previous work data in which normal hepcidin levels were reported, and it can be explained by using of mean values of concentrations of all patients to present data. Indeed, also our results do not show significant differences in Hepcidin levels between EPP and controls, probably due to the large dispersion of the values among the mean value. In contrast, the stratification on sex and microcytosis shows the increased hepcidin levels in patients without iron deficiency. However, the low number of patients did not allow us to calculate the statistical significance.

In addition, no correlation was found between MCV and ferritin, TfS%, and iron values in our experiments, confirming that microcytosis does not directly depend on iron deficiency. In support of these observations, the study by Lyoumi et al. on iron metabolism in Fech^m1pas^ mice showed how a FECH deficiency leads to microcytic anemia, despite normal serum levels of iron, ferritin, and Hepcidin mRNA (Lyoumi et al., 2007). In the same paper, the authors showed a positive correlation between erythrocyte PPIX and serum transferrin (Tf) levels suggesting that a redistribution between peripheral iron stores, the spleen, and the bone marrow occurred in EPP to meet erythropoiesis requirement.

Instead, an inverse correlation between PPIX values and MCV has been observed in our population and mean PPIX values are reported significantly higher in male and female microcytic patients than in normocytic ones. In the light of this information, it is possible to hypothesize either an active role of the accumulation of PPIX in the reduction of MCV through a probable toxic action on the cell membrane of the red blood cells or that microcytosis results from a heme deficiency. The first hypothesis is not conceivable since signs of hemolysis have never been observed in EPP. On the other hand, the severity of heme deficiency directly depends on the residual activity of the FECH enzyme and subsequent PPIX accumulation. The latter feature could also be further increased by heme biosynthesis stimulation in a vicious circle. Unfortunately, neither the degree of FECH deficiency nor the quantification of heme is available for our patients to support this hypothesis. At the same time, the amount of PPIX could also act as a sensor of FECH deficiency modulating the intestinal iron absorption through the hepcidin-dependent pathway in conditions of repleted iron stores and normal hemoglobinization. This suggestion is supported by the observation that an inverse correlation was reported between erythrocyte PPIX and serum iron levels (Delaby et al., 2009). Moreover, Barmann et al. confirmed that the reduced FECH activity in EPP patients was sufficient to increase the transcription of ALAS2 mRNA (Barman-Aksozen et al., 2019). As long as iron is kept at a very low concentration, the increase in ALAS2 mRNA will not result in an increase in ALAS2 protein because of the iron-mediated translational repression. This could explain the protective role of iron deficiency in EPP despite its dual opposite effect in the heme synthesis regulation. Indeed, the iron cannot be used by abnormal FECH to form heme in these patients, but it became beneficial in preventing further PPIX accumulation (Poli et al., 2021).

However, when an iron deficiency occurs, hemoglobin synthesis levels should be regulated by factors other than PPIX. The precise role of PPIX in causing microcytosis in EPP patients remains to be established by further investigations. Whether hepcidin levels are dysregulated by increased levels of PPIX or by other abnormalities in EPP is currently unknown.

## Data Availability Statement

The raw data supporting the conclusions of this article will be made available by the authors, without undue reservation.

## Ethics Statement

The studies involving human participants were reviewed and approved by Comitato di Etica Milano area 2. The patients/participants provided their written informed consent to participate in this study.

## Author Contributions

GG: conceptualization. ED and FG: writing—original draft preparation. LD: biochemical experiments. LD and FG: statistical analysis. VB: molecular diagnosis. GD and AD: patients visit. VB, ED, and GG: writing—review editing. IN: visualization. All authors contributed to the article and approved the submitted version.

## Funding

This study was funded in part by the Italian Ministry of Health (RC2021 and RC2022).

## Conflict of Interest

The authors declare that the research was conducted in the absence of any commercial or financial relationships that could be construed as a potential conflict of interest.

## Publisher’s Note

All claims expressed in this article are solely those of the authors and do not necessarily represent those of their affiliated organizations, or those of the publisher, the editors and the reviewers. Any product that may be evaluated in this article, or claim that may be made by its manufacturer, is not guaranteed or endorsed by the publisher.

## References

[ref1] Anstey A. V. Hift R. J. (2007). Liver disease in erythropoietic protoporphyria: insights and implications for management. Gut 56, 1009–1018. doi: 10.1136/gut.2006.097576, PMID: 17360790 PMC1994365

[ref2] Balwani M. (2019). Erythropoietic Protoporphyria and X-linked Protoporphyria: pathophysiology, genetics, clinical manifestations, and management. Mol. Genet. Metab. 128, 298–303. doi: 10.1016/j.ymgme.2019.01.020, PMID: 30704898 PMC6656624

[ref001] Balwani M. Naik H. Anderson K. E. Bissell D. M. Bloomer J. Bonkovsky H. L.. (2017). Clinical, biochemical, and genetic characterization of North American patients with erythropoietic protoporphyria and X-linked protoporphyria. JAMA Dermatol. 153, 789–796. doi: 10.1001/jamadermatol.2017.1557, PMID: 28614581 PMC5710403

[ref007] Barman-Aksozen J. Beguin C. Dogar A. M. Schneider-Yin X. Minder E. I. (2013). Iron availability modulates aberrant splicing of ferrochelatase through the iron- and 2-oxoglutarate dependent dioxygenase Jmjd6 and U2AF^65^. Blood Cells Mol. Dis. 51, 151–161. doi: 10.1016/j.bcmd.2013.05.008, PMID: 23787363

[ref3] Barman-Aksoezen J. Girelli D. Aurizi C. Schneider-Yin X. Campostrini N. Barbieri L.. (2017). Disturbed iron metabolism in erythropoietic protoporphyria and association of GDF15 and gender with disease severity. J. Inherit. Metab. Dis. 40, 433–441. doi: 10.1007/s10545-017-0017-7, PMID: 28185024

[ref4] Barman-Aksozen J. Halloy F. Iyer P. S. Schumperli D. Minder A. E. Hall J.. (2019). Delta-aminolevulinic acid synthase 2 expression in combination with iron as modifiers of disease severity in erythropoietic protoporphyria. Mol. Genet. Metab. 128, 304–308. doi: 10.1016/j.ymgme.2019.04.013, PMID: 31076252

[ref5] Bossi K. Lee J. Schmeltzer P. Holburton E. Groseclose G. Besur S.. (2015). Homeostasis of iron and hepcidin in erythropoietic protoporphyria. Eur. J. Clin. Investig. 45, 1032–1041. doi: 10.1111/eci.12503, PMID: 26199063

[ref008] Buzzetti E. Ventura P. Corradini E. (2022). Iron in porphyrias: friend or foe? Diagnostics 12:272. doi: 10.3390/diagnostics12020272, PMID: 35204362 PMC8870839

[ref6] Chiara M. Primon I. Tarantini L. Agnelli L. Brancaleoni V. Granata F.. (2020). Targeted resequencing of FECH locus reveals that a novel deep intronic pathogenic variant and eQTLs may cause erythropoietic protoporphyria (EPP) through a methylation-dependent mechanism. Genet. Med. 22, 35–43. doi: 10.1038/s41436-019-0584-0, PMID: 31273344

[ref7] Cook J. D. Finch C. A. (1979). Assessing iron status of a population. Am. J. Clin. Nutr. 32, 2115–2119. doi: 10.1093/ajcn/32.10.2115, PMID: 484529

[ref8] Delaby C. Lyoumi S. Ducamp S. Martin-Schmitt C. Gouya L. Deybach J. C.. (2009). Excessive erythrocyte PPIX influences the hematologic status and iron metabolism in patients with dominant erythropoietic protoporphyria. Cell Mol. Biol. 55, 45–52. PMID: 19268001

[ref002] Di Pierro E. Granata F. De C. M. Rossi M. Ricci A. Marcacci M.. (2022). Recognized and emerging features of erythropoietic and X-linked protoporphyria. Diagnostics 12:151. doi: 10.3390/diagnostics12010151, PMID: 35054318 PMC8775248

[ref009] Di Pierro E. De Canio M. Mercadante R. Savino M. Granata F. Tavazzi D.. (2021). Laboratory diagnosis of porphyria. Diagnostics 11:1343. doi: 10.3390/diagnostics11081343, PMID: 34441276 PMC8391404

[ref9] Fleming R. E. Sly W. S. (2001). Hepcidin: a putative iron-regulatory hormone relevant to hereditary hemochromatosis and the anemia of chronic disease. Proc. Natl. Acad. Sci. U. S. A. 98, 8160–8162. doi: 10.1182/blood-2006-04-014142, PMID: 11459944 PMC37412

[ref10] Ganz T. (2013). Systemic iron homeostasis. Physiol. Rev. 93, 1721–1741. doi: 10.1152/physrev.00008.2013, PMID: 24137020

[ref11] Gouya L. Puy H. Robreau A. M. Bourgeois M. Lamoril J. Da S.. (2002). The penetrance of dominant erythropoietic protoporphyria is modulated by expression of wildtype FECH. Nat. Genet. 30, 27–28. doi: 10.1038/ng809, PMID: 11753383

[ref12] Holme S. A. Worwood M. Anstey A. V. Elder G. H. Badminton M. N. (2007). Erythropoiesis and iron metabolism in dominant erythropoietic protoporphyria. Blood 110, 4108–4110. doi: 10.1182/blood-2007-04-088120, PMID: 17804693

[ref13] Karim Z. Lyoumi S. Nicolas G. Deybach J. C. Gouya L. Puy H. (2015). Porphyrias: A 2015 update. Clin. Res. Hepatol. Gastroenterol. 39, 412–425. doi: 10.1016/j.clinre.2015.05.009, PMID: 26142871

[ref14] Lecha M. Puy H. Deybach J. C. (2009). Erythropoietic protoporphyria. Orphanet J. Rare Dis. 4:19. doi: 10.1186/1750-1172-4-19, PMID: 19744342 PMC2747912

[ref15] Lyoumi S. Abitbol M. Andrieu V. Henin D. Robert E. Schmitt C.. (2007). Increased plasma transferrin, altered body iron distribution, and microcytic hypochromic anemia in ferrochelatase-deficient mice. Blood 109, 811–818. doi: 10.1182/blood-2006-04-014142, PMID: 17003376

[ref003] Poli A. Schmitt C. Moulouel B. Mirmiran A. Puy H. Lefebvre T.. (2021). Iron, heme synthesis and erythropoietic porphyrias: a complex interplay. Metabolites 11:798. doi: 10.3390/metabo11120798 34940556 PMC8705723

[ref005] Silva B. Faustino P. (2015). An overview of molecular basis of iron metabolism regulation and the associated pathologies. Biochim. Biophys. Acta 1852, 1347–1359. doi: 10.1016/j.bbadis.2015.03.011, PMID: 25843914

[ref16] Skikne B. S. Flowers C. H. Cook J. D. (1990). Serum transferrin receptor: a quantitative measure of tissue iron deficiency. Blood 75, 1870–1876. doi: 10.1182/blood.V75.9.1870.1870, PMID: 2331526

[ref006] Taketani S. Adachi Y. Nakahashi Y. (2000). Regulation of the expression of human ferrochelatase by intracellular iron levels. Eur. J. Biochem. 267, 4685–4692. doi: 10.1046/j.1432-1327.2000.01519.x, PMID: 10903501

[ref17] Tanno T. Noel P. Miller J. L. (2010). Growth differentiation factor 15 in erythroid health and disease. Curr. Opin. Hematol. 17, 184–190. doi: 10.1097/MOH.0b013e328337b52f, PMID: 20182355 PMC2884377

[ref18] Wensink D. Coenen S. Wilson J. H. P. Wagenmakers M. A. E. M. Langendonk J. G. (2021). Liver involvement in patients with erythropoietic protoporphyria. Dig. Liver Dis. 56:151859. doi: 10.1016/j.anndiagpath.2021.151859, PMID: 34475006

[ref004] Wilkinson N. Pantopoulos K. (2014). The IRP/IRE system *in vivo*: insights from mouse models. Front. Pharmacol. 5:176. doi: 10.3389/fphar.2014.00176, PMID: 25120486 PMC4112806

